# Unusual Presentation of Orofacial Granulomatosis Leading to the Incidental Diagnosis of Idiopathic Pulmonary Fibrosis: A Case Report

**DOI:** 10.7759/cureus.108767

**Published:** 2026-05-13

**Authors:** Farouk Mohammed, Oussama Bentahar

**Affiliations:** 1 Odontology Department, Faculty of Medicine, Pharmacy, and Dentistry, Sidi Mohamed Ben Abdellah University, Fes, MAR

**Keywords:** clinical case report, crohn’s disease (cd), orofacial granulomatosis, pulmonary fibrosis, pulmonary sarcoidosis

## Abstract

Orofacial granulomatosis (OFG) is a rare chronic inflammatory disorder characterized by non-caseating granulomatous inflammation involving the oral and maxillofacial region. Its heterogeneous clinical presentation and overlap with systemic granulomatous diseases often make diagnosis challenging. We report the case of a 54-year-old woman presenting with a four-month history of progressive mandibular mucosal hypertrophy mimicking an epulis-like lesion. Clinical examination revealed multiple pedunculated, firm, painless mucosal lesions localized to the anterior mandibular ridge in a fully edentulous patient, without lip swelling or fissured tongue. Histopathological analysis demonstrated non-caseating granulomatous inflammation composed of epithelioid histiocytes, lymphocytic infiltrates, and Langhans-type multinucleated giant cells.

A comprehensive multidisciplinary evaluation was performed to exclude systemic granulomatous diseases, including Crohn’s disease, sarcoidosis, hypersensitivity reactions, infectious diseases, and Melkersson-Rosenthal syndrome. High-resolution computed tomography (HRCT) revealed bilateral basal and subpleural reticulations, architectural distortion, and ground-glass opacities consistent with a probable usual interstitial pneumonia (UIP) pattern. Pulmonary function testing demonstrated a restrictive ventilatory defect with reduced diffusing capacity of the lungs for carbon monoxide (DLCO). Following multidisciplinary assessment and the exclusion of alternative etiologies, a diagnosis of idiopathic pulmonary fibrosis (IPF) was established according to American Thoracic Society/European Respiratory Society (ATS/ERS) criteria. This finding was considered incidental and unrelated to the oral lesions.

Management included systemic corticosteroid therapy, followed by surgical excision of the fibrotic lesions using an electrosurgical approach to facilitate prosthetic rehabilitation. Postoperative evolution was favorable, with no recurrence after two years of follow-up, although pulmonary function progressively deteriorated due to IPF.

This case highlights the diagnostic challenges associated with atypical OFG presentations and emphasizes the importance of a rigorous multidisciplinary approach to exclude systemic diseases while avoiding inappropriate causal associations.

## Introduction

Orofacial granulomatosis (OFG) is an uncommon chronic inflammatory disorder characterized by non-caseating granulomatous inflammation involving the oral and maxillofacial region [1]. Although its pathogenesis remains incompletely understood, infectious, genetic, immunological, and hypersensitivity-related mechanisms have been proposed [2]. OFG remains a rare entity with poorly established prevalence due to its heterogeneous clinical spectrum and overlap with several systemic granulomatous diseases [3].

The term “idiopathic OFG” is generally reserved for granulomatous lesions confined to the orofacial region in the absence of systemic diseases such as Crohn’s disease or sarcoidosis, as well as syndromic conditions including Melkersson-Rosenthal syndrome [3,4]. Clinically, OFG most commonly presents as persistent lip swelling; however, ulcerative, hypertrophic, and atypical presentations have also been reported, potentially leading to delayed diagnosis and therapeutic difficulties [5].

Because histopathological findings are not pathognomonic, diagnosis requires a structured exclusion process integrating clinical, histological, radiological, and multidisciplinary evaluation to rule out infectious, inflammatory, autoimmune, and systemic granulomatous disorders [4,6]. Previous case reports have highlighted the complexity of OFG diagnosis due to atypical clinical manifestations, overlap with systemic diseases, and the absence of standardized therapeutic protocols [7].

We report an unusual presentation of OFG manifesting as mandibular mucosal hypertrophy mimicking an epulis-like lesion. A comprehensive diagnostic workup led to the incidental identification of idiopathic pulmonary fibrosis (IPF). This report underscores the importance of multidisciplinary assessment in atypical OFG presentations and was prepared in accordance with the CARE guidelines [8].

## Case presentation

A 54-year-old woman was referred to the department of oral surgery for the evaluation of multiple hypertrophic mucosal lesions involving the anterior mandibular ridge, with a four-month history of progressive enlargement. Her medical history was unremarkable, and no prior treatment had been administered.

Extraoral examination revealed no lymphadenopathy, facial asymmetry, or facial nerve impairment. Intraoral examination demonstrated multiple pedunculated, firm, painless mucosal lesions of normal coloration, measuring approximately 0.8-1.5 cm in greatest dimension, localized to the anterior mandibular region. The patient was fully edentulous, with normal tongue and lip morphology, without fissured tongue or evidence of granulomatous cheilitis (Figure 1A-1C).

**Figure 1:**
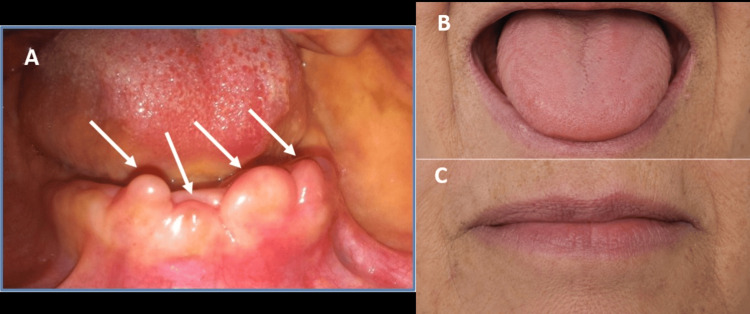
Clinical examination (A) Multiple pedunculated hypertrophic mucosal lesions measuring approximately 0.8-1.5 cm in greatest dimension, localized in the anterior mandibular region in a fully edentulous patient. (B) Normal tongue appearance without fissuring or evidence of granulomatous involvement. (C) Normal lip morphology without swelling or evidence of granulomatous cheilitis

Panoramic radiography showed no underlying osseous abnormalities. The panoramic radiograph confirmed the absence of osseous involvement or impacted lesions associated with the mandibular mucosal hypertrophy (Figure 2). The initial clinical impression suggested an epulis-like lesion, prompting an incisional biopsy prior to definitive management.

**Figure 2:**
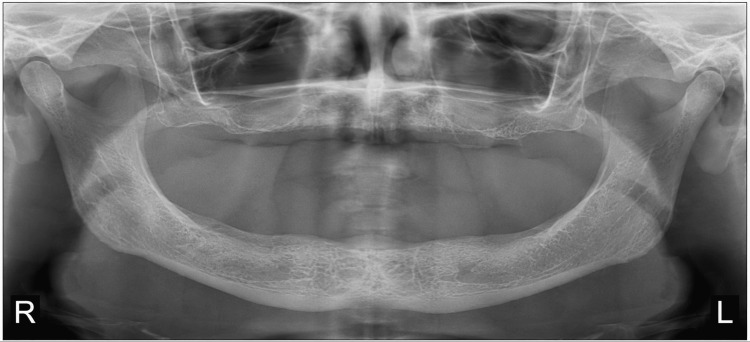
Panoramic radiograph Panoramic radiography showing complete edentulism without underlying osseous abnormalities or radiographic evidence of mandibular bone involvement. “R” and “L” indicate the patient’s right and left sides, respectively

Histopathological examination revealed non-caseating granulomatous inflammation composed of epithelioid histiocytes, lymphocytic infiltrates, and Langhans-type multinucleated giant cells (Figure 3). The absence of caseous necrosis, combined with epithelioid granulomas and multinucleated giant cells, supported a noninfectious granulomatous process.

**Figure 3:**
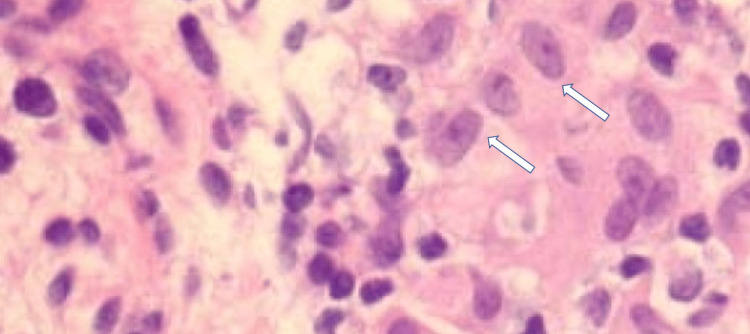
Histological examination Non-caseating granulomatous inflammation composed of epithelioid histiocytes, lymphocytic infiltrates, and Langhans-type multinucleated giant cells is indicated by arrows

These findings supported the presence of granulomatous disease, leading to a structured differential diagnostic workup. The principal considerations included systemic granulomatous disorders, OFG, and Melkersson-Rosenthal syndrome. The latter was excluded due to the absence of facial paralysis and fissured tongue. Granulomatous cheilitis was ruled out based on clinical findings and a minor salivary gland biopsy, which showed no granulomatous involvement. The diagnosis of OFG was retained after the exclusion of systemic granulomatous diseases and syndromic conditions.

To exclude systemic etiologies, a comprehensive evaluation was undertaken. Gastroenterological assessment, including imaging and stool analysis, was unremarkable, excluding Crohn’s disease. Allergological testing revealed no hypersensitivity.

The patient was subsequently referred for pulmonological evaluation to investigate possible sarcoidosis. Laboratory tests, including serum calcium, renal function, and urinalysis, were within normal limits. High-resolution computed tomography (HRCT) did not demonstrate hilar or mediastinal lymphadenopathy. However, it revealed bilateral basal and subpleural reticulations, architectural distortion, and ground-glass opacities (Figure 4A-4D), consistent with a probable usual interstitial pneumonia (UIP) pattern.

**Figure 4:**
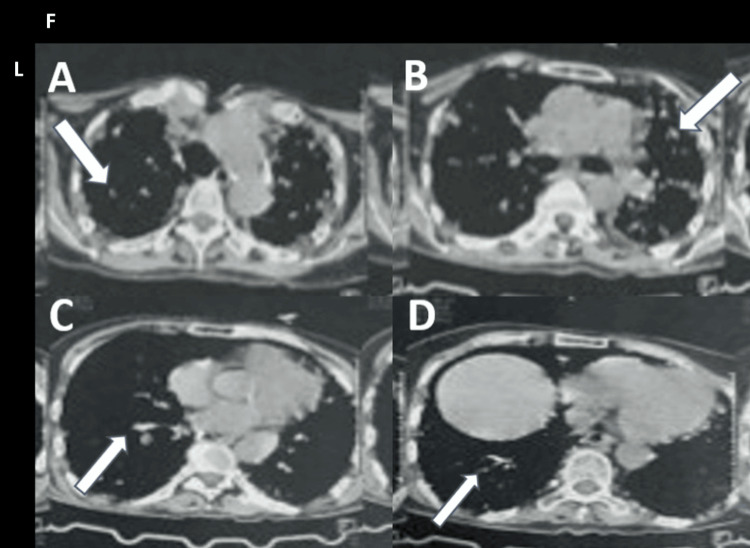
High-resolution computed tomography (HRCT) (A-D) Bilateral axial HRCT images at different levels. It revealed subpleural reticulations, architectural distortion, and ground-glass opacities. Arrows indicate areas of interstitial involvement. “F” indicates the frontal orientation, and “L” indicates the patient’s left side

Pulmonary function tests demonstrated a restrictive ventilatory defect with reduced diffusing capacity of the lungs for carbon monoxide (DLCO) (Table 1). A multidisciplinary discussion involving pulmonology, radiology, and internal medicine excluded sarcoidosis and other causes of interstitial lung disease, including autoimmune, infectious, cardiac, drug-induced, and neoplastic conditions.

**Table 1:** Pulmonary diffusion parameters Pulmonary diffusion testing revealed a marked reduction in DLCO (46% predicted) and DLCO/VA (59% predicted), indicating impaired alveolar-capillary gas transfer. The decreased alveolar volume (VA, 65% predicted) supports an underlying restrictive ventilatory pattern. Hemoglobin level was within normal limits and does not account for the reduced diffusion capacity DLCO, diffusing capacity of the lungs for carbon monoxide; KCO, carbon monoxide transfer coefficient; VA, alveolar volume; Hb, hemoglobin

Parameter	Patient Value	Predicted Value	Percent Predicted	Unit	Reference Range
DLCO	9.2	20.1	46%	mL/minute/mmHg	80%-120% predicted
DLCO/VA (KCO)	2.7	4.6	59%	mL/minute/mmHg/L	75%-125% predicted
VA	3.40	5.20	65%	L	80%-120% predicted
Hb	13.2	-	-	g/dL	13-17 (male); 12-16 (female)

In accordance with current American Thoracic Society/European Respiratory Society (ATS/ERS) criteria, a diagnosis of idiopathic pulmonary fibrosis was established. This finding was considered incidental and unrelated to the oral lesions.

Following the exclusion of systemic granulomatous diseases, a diagnosis of OFG was retained, supported by compatible clinical and histopathological findings in the absence of an identifiable systemic or syndromic cause.

Management addressed both conditions independently. Antifibrotic therapy with nintedanib (150 mg twice daily) was initiated for IPF, with satisfactory adherence and initial tolerance.

For OFG, systemic corticosteroid therapy with betamethasone (2 mg/day for seven days) was administered. Due to the fibrotic and well-demarcated nature of the lesions and limited response to medical therapy, surgical excision was performed one month later using an electrosurgical approach to facilitate prosthetic rehabilitation (Figure 5). Postoperative care included amoxicillin (2 g/day for eight days), paracetamol (1 g every six hours as needed), and chlorhexidine mouth rinses (0.12%) twice daily for seven days.

**Figure 5:**
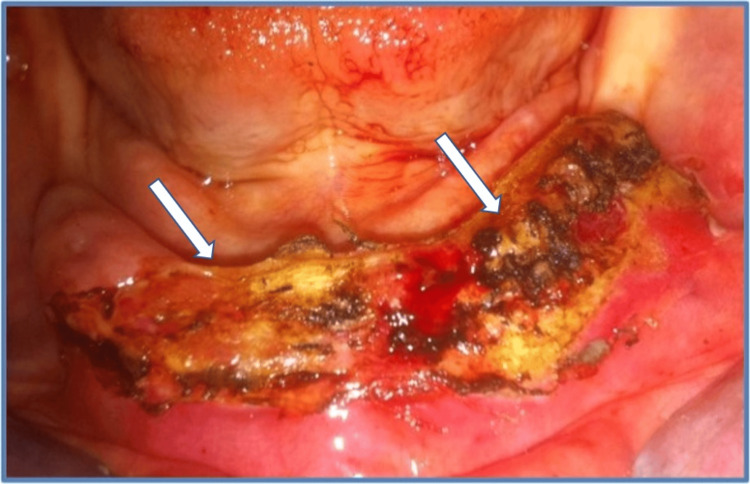
Surgical management Surgical excision with an electrosurgical approach. Arrows indicate the surgical site

After 15 days of antifibrotic therapy, the patient developed clinical worsening characterized by a persistent chronic cough, suggestive of IPF exacerbation. Systemic corticosteroid therapy with betamethasone was therefore introduced using a tapering regimen: 2 mg/day for one week, followed by 1.5 mg/day, 1 mg/day, and 0.5 mg/day, each for one week. This regimen was maintained over one month, resulting in progressive clinical improvement.

The patient reported high satisfaction with the therapeutic outcome of OFG, particularly following prosthetic rehabilitation, which significantly improved oral function.

At the two-year follow-up, no recurrence of oral lesions was observed. However, pulmonary function progressively declined, ultimately requiring long-term oxygen therapy.

## Discussion

Orofacial granulomatosis is an uncommon inflammatory disorder with a female predominance, typically occurring between the second and third decades of life, although cases outside this range have been reported [1]. The term “idiopathic OFG” is reserved for cases strictly confined to the oral cavity in the absence of systemic disease [6].

Classically, OFG presents with localized swelling or ulceration [7]. In the present case, mucosal hypertrophy mimicking an epulis constituted an atypical presentation, illustrating the diagnostic challenges associated with this condition. Previous case reports have highlighted several diagnostic and therapeutic complexities associated with OFG, including atypical clinical presentations, delayed diagnosis, overlap with systemic granulomatous diseases, and the absence of standardized therapeutic protocols [9].

Histopathological examination remains indispensable, with characteristic findings of non-caseating granulomas composed of epithelioid histiocytes and multinucleated giant cells [5]. However, these features are not pathognomonic and must be interpreted within the broader clinical and investigative context.

Diagnosis relies on a structured exclusion process, incorporating clinical evaluation and targeted investigations to rule out systemic granulomatous diseases such as Crohn’s disease, sarcoidosis, and hypersensitivity reactions [4,9].

In this case, a comprehensive stepwise approach enabled the exclusion of these conditions. The absence of neurological signs ruled out Melkersson-Rosenthal syndrome, while clinical and histological findings excluded granulomatous cheilitis.

The identification of pulmonary fibrosis was considered incidental, and no direct etiological relationship between OFG and idiopathic pulmonary fibrosis could be established in the present case. Particular caution is required when interpreting such associations in isolated case reports to avoid implying causality. According to ATS/ERS guidelines, the diagnosis of IPF is based on the integration of clinical, functional, and radiological findings following the exclusion of other causes of interstitial lung disease. HRCT typically demonstrates a UIP pattern, and pulmonary function tests reveal a restrictive defect with reduced DLCO. Multidisciplinary evaluation remains essential for diagnostic confirmation [9,10].

In the present case, these criteria were fulfilled, supporting the diagnosis of IPF.

The therapeutic management of OFG remains non-standardized; however, corticosteroids constitute the mainstay of treatment [9]. Surgical intervention may be indicated in fibrotic lesions. In this patient, combined medical and surgical management was tailored to lesion characteristics.

To the best of our knowledge, this is a rare presentation of OFG mimicking an epulis and leading to the incidental diagnosis of idiopathic pulmonary fibrosis, which has seldom been reported in the literature.

## Conclusions

Orofacial granulomatosis is a rare and diagnostically challenging entity with variable clinical presentation. Atypical forms may mimic reactive or neoplastic lesions, potentially delaying diagnosis and management. Accurate diagnosis requires the systematic exclusion of infectious, inflammatory, autoimmune, and systemic granulomatous diseases through multidisciplinary evaluation. This case highlights the importance of integrating clinical, histopathological, radiological, and functional findings in the diagnostic process while carefully avoiding inappropriate causal interpretation between OFG and incidental systemic conditions such as idiopathic pulmonary fibrosis.

## References

[1] Shetty N, Subramanyam RV, Srikanth G, Solomon MC (2021). Orofacial granulomatosis: a case report and a proposal of a diagnostic algorithm for oral granulomatous lesions. J Oral Maxillofac Pathol.

[2] Guillaume F, Maria P, Jocelyn C, Sara L, Sarah C (2018). Management of orofacial granulomatosis: a case report. J Oral Med Oral Surg.

[3] Troiano G, Dioguardi M, Giannatempo G (2015). Orofacial granulomatosis: clinical signs of different pathologies. Med Princ Pract.

[4] Miest R, Bruce A, Rogers RS 3rd (2016). Orofacial granulomatosis. Clin Dermatol.

[5] Archibald CW, Punja KG, Oryschak AF (2012). Orofacial granulomatosis presenting as bilateral eyelid swelling. Saudi J Ophthalmol.

[6] Tilakaratne WM, Freysdottir J, Fortune F (2008). Orofacial granulomatosis: review on aetiology and pathogenesis. J Oral Pathol Med.

[7] Badshah MB, Walayat S, Ahmed U, Dhillon S, Yong S, Kane S, Thievanayagam S (2017). Treatment of orofacial granulomatosis: a case report. J Med Case Rep.

[8] Gagnier JJ, Kienle G, Altman DG, Moher D, Sox H, Riley D (2014). The CARE guidelines: consensus-based clinical case report guideline development. J Clin Epidemiol.

[9] Sopi Krasniqi M, Sllamniku Dalipi Z, Kastrati Dragidella D, Kondirolli L (2023). Treatment of orofacial granulomatosis-9-month follow-up: a case report. Clin Med Insights Case Rep.

[10] Bandyopadhyay D, Mirsaeidi MS (2023). Sarcoidosis-associated pulmonary fibrosis: joining the dots. Eur Respir Rev.

